# Playing video games in community spaces and adolescent loneliness: a cross-sectional study

**DOI:** 10.1186/s13034-025-00980-8

**Published:** 2025-11-05

**Authors:** Ariadna Corbella-González, Alicia Cal-Herrera, Olga I. Fernández-Rodríguez

**Affiliations:** Department of Health Sciences, INPRO, European University Miguel de Cervantes, C. del Padre Julio Chevalier, 2, Valladolid, 47012 Spain

**Keywords:** Videogames, Loneliness, Community, Adolescence, Mental health, Occupational participation

## Abstract

**Background:**

Loneliness is currently very prevalent among adolescents, negatively affecting their physical, mental, and social health. Playing videogames is one of the most common leisure activities, which has been associated with multiple negative and positive outcomes for mental health. Depending on their use, they can generate mental and social health benefits or lead to an addiction that disrupts daily life and affects the adolescent’s mental health. Community spaces for young people create safe environments where adolescents can gain access to different meaningful leisure activities, but the association of community videogame play with feelings of loneliness has not been researched to this date. Therefore, the purpose of this study is to understand the feelings of loneliness among adolescents aged 14 to 20 who attend community spaces to play videogames.

**Methods:**

This descriptive cross-sectional study included adolescents aged between 14 and 20. A sociodemographic questionnaire, questions related to videogame use, and the Richaud de Minzi and Sacchi Adolescent Loneliness Scale were administered.

**Results:**

A total of 112 adolescents with a mean age of 18.21 years (± 1.52) were included. It was found that adolescents who played alone had a worse perception of their friendships, family ties, themselves, and their social and adaptive skills, compared to adolescents who exclusively played when accompanied.

**Conclusions:**

Participation in meaningful activities, such as shared videogame use within community resources, may decrease the likelihood of developing feelings of loneliness in the adolescent population.

## Background

### Feelings of loneliness in adolescence

Adolescence is a critical stage of human development where physical, psychological, and social health problems that emerge can persist into adulthood and affect lifestyle [1, 2].

The personal and emotional evolution resulting from coping with these challenges is influenced by the adolescent’s psychosocial factors, where social environment plays a fundamental role. This can act as a protective factor when adolescents receive affection, support, supervision, and communication, or become a risk factor when there is a disconnection from the environment, which can compromise vital development and generate feelings of loneliness [3, 4].

Loneliness has become a social problem of growing concern among adolescents, both because of its prevalence and because of its implications for mental health and overall well-being in this context. As defined by Perlman & Peplau, loneliness is traditionally known as *“the unpleasant experience that occurs when a person’s social network is deficient in some important aspect*,* whether in quantity or quality*,* and that results from the discrepancy between desired and achieved levels of social relationships”* [5].

This discrepancy is particularly significant during adolescence, as this stage tends to generate high and unrealistic expectations about social relationships, while at the same time the brain is undergoing major developmental changes that increase sensitivity to social feedback, making adolescents especially vulnerable to feelings of exclusion or disconnection [4].

Despite being a generation that has greater opportunities for social interaction due to technological advances and greater openness to emotional expression in society, recent research shows a high prevalence of loneliness among youth, with a national study of spanish youth aged 16 to 29 reporting that one in four young people feel lonely [6].

The experience of loneliness is subjective and varies from individual to individual, making it difficult to identify a profile more prone to these feelings. However, evidence suggests an association between loneliness and youth, involving various factors such as socioeconomic difficulties, low satisfaction with family and friendship relationships, the presence of mental or physical disorders, certain psychological and personality characteristics, as well as being in an unfavourable social environment or having experienced adverse situations during childhood [7].

### Videogames: benefits and risks

Playing videogames has become one of the most significant leisure activities for adolescents, with 48.8% of spanish adolescents playing videogames every day [8]. A habit that could be capable of promoting social participation and becoming a tool that reduces loneliness if engaged appropriately [9].

This activity encompasses a wide range of gaming experiences that cater to diverse preferences, enabling engagement with varied gameplay formats through solo play or multiplayer modes that could involve both online or physical co-play. Notably, games that promote playing with friends tend to be perceived as more enjoyable than solitary gaming experiences or interactions with unknown players online [10]. Although this activity has been associated with general benefits like developing stress coping mechanisms, strengthening attentional capabilities, vision improvement, happiness and prosocial and civic behaviours [11].

The videogames that offer multiplayer choices or that enable online interaction with other players could become a source of social support for adolescents, offering them the opportunity to expand their network of social relationships with people who share similar interests and strengthen their sense of belonging to a group [12]. Furthermore, evidence supports its enriching and multifaceted nature at this vital stage when used in a balanced manner [9, 13–15].

However, data from spanish adolescents aged 11 to 18 reported 8.45% of the 946 participants met the criteria for addiction, while 52.70% exhibited moderate symptoms for Internet Gaming Disorder [16], indicating that there is a percentage of adolescents who use videogames in an uncontrolled manner, causing disruptions in both their daily routines and functioning at the psychological, family, social, work, school, and behavioral levels [17, 18].

At the onset of addiction, psychosocial functioning is a key factor influencing the severity of the impact on daily life caused by uncontrolled videogame use. For that reason, it will be necessary to consider the context and environments where adolescents engage in this activity [19–21].

There may be underlying personal situations that cause psychological distress in adolescents, triggering the use of videogames as a form of escape within the school or family context [22], while the amount of time spent playing and the people involved in the game influence the onset of addiction [23]. Therefore, adolescents with uncontrolled access who play alone are at greater risk of developing disorders associated with problematic videogame use, unlike those who play in shared environments and have greater social participation face-to-face or online [24].

### The role of community resources tailored to young people

From a community perspective, providing environments where adolescents can develop safely prevents the emergence of social risk factors linked to the development of addiction [21].

Community resources provide different programs and activities structured around young people’s needs. Young people’s experiences show that they value these centres for the opportunity they provide to exercise their autonomy in choosing activities, the sense of belonging and community they promote, the freedom of personal expression, and the feeling of security that comes from having an environment they can freely turn to [25].

The use of videogames within these community resources and its implications for feelings of loneliness has been an underexplored issue. While there are some studies that have analysed the benefits of playing online with other people in the COVID-19 context [26, 27], or focused on the social participation in videogames of people with conditions such as autism spectrum disorder or spinal cord injury [28, 29], the role of local community resources in loneliness has never before been considered within this activity. Therefore, the aim of this research was to understand the feelings of loneliness experienced by adolescents aged 14 to 20 who attend community spaces to play videogames.

## Methods

### Participants

This study had a descriptive cross-sectional design and was conducted in accordance with the STROBE guidelines (*Strengthening the Reporting of Observational Studies in Epidemiology*) [30].

The inclusion criteria were being between 14 and 20 years old and playing videogames as a leisure activity in community spaces, whereas the exclusion criteria were being outside the specified age range or lacking interest in playing videogames. Prior to answering the questionnaire, participants were asked to fill out an informed consent. They were also briefed on the study’s objectives and assured of the anonymity of their answers.

### Sample size calculation

The G*power program was used to calculate the sample size, establishing a confidence level of 95% (alpha risk of 0.05) and an accuracy of 10% in a two-tailed test, considering an estimated proportion of 0.5 and a replacement rate of 1%, obtaining a minimum sample size of 97 subjects.

Convenience sampling was used for the recruitment of participants, with a total of 156 individuals invited to participate in the study during August and September 2024. In the end, 112 participants agreed to take part, resulting in a 71.8% participation rate.

### Procedure

Participants for this study were sought out from community youth centres that had videogame facilities available for free use. An agreement was established with the centres allowing the principal investigator to attend during the designated data collection period and approach adolescents to invite them to participate in the study. It was emphasized at all times that participation was voluntary and that they could freely decide whether or not to complete the questionnaire.

Adolescents that agreed to participate in the study were informed of their rights and anonymity and were asked to fill out an informed consent. This consent was signed by their parents or legal guardians in the case of underage participants or by themselves if they were over 18 years old. The participants were then asked to fill out the questionnaire and to ask any questions that they needed during the process.

Questionnaires were collected and kept under the care of the main researcher for this investigation.

### Videogames and community centers

In this study, videogames are broadly defined to include console, mobile, and tablet formats, reflecting the range of platforms available to participants within the community centers, as adolescents could access videogames through the consoles provided by the centers or by bringing their own portable consoles, tablets, or mobile devices.

The community centers included in the study are free public facilities that operate daily and are staffed by trained adults who supervise the space and provide both structured programs and opportunities for unstructured free time for youth aged 14 to 30 years. These centers are primarily located in urban areas and offer spaces for multiple activities (e.g., music rehearsals, dance, studying), in addition to videogame use. Videogaming is incorporated into daily operations either as unstructured recreational time using various consoles or as occasional organized sessions.

With regard to videogames, specific rules regulate console access, such as a maximum playtime of two hours and the requirement that at least two players be present to rent a console. Adolescents may still engage in solitary play within the centers or access online games through their own portable consoles or mobile phones, but physical co-play is actively encouraged.

### Assessment measures

The Adolescent Loneliness Scale was administered through an interview with the participants. This scale is a self-administered assessment tool that has been used in adolescents, showing adequate psychometric properties in this population group [31]. It consists of 32 items which measure feelings of loneliness using a 5-point Likert scale based on four areas:*“Peer rejection”*, *“Family deficits and parental rejection”*,* “Personal inadequacy”* and “*Lack of social skills and significant separation”* [32]. Each of these areas includes eight items, with scores ranging from 8 to 40 points. There is no cutoff point, but higher scores are considered to indicate a greater perception of loneliness within the area to which they belong.

The reliability for the loneliness scale overall given in Cronbach’s alpha for this study was 0.91, with the coefficients 0.85 for peer rejection, 0.90 for family deficits, 0.70 for personal inadequacy and 0.70 for lack of social skills and significant separation. These figures are similar to those found previously in other studys, with coefficients of 0.89 for peer rejection, 0.88 for family deficits, 0.78 for personal inadequacy, and 0.75 for lack of social skills and significant separation from a sample of 1319 adolescents aged 12 to 15 years old [31], or data of 0.90, 0.88, 0.78, and 0.79, respectively, from 241 adolescents between the ages of 11 and 15 [33].

In addition, a sociodemographic section was included with variables such as age, gender, and education, along with various questions related to videogame use based on different questionnaires [34]. The questions about videogames focused on different aspects of the adolescent’s occupational participation, which were related to: the amount of time spent playing per day, the days per week they play, and the people they usually play with.

### Statistical analysis

A quantitative study was conducted using version 24 of the IBM SPSS statistical software (SPSS Inc.). The normality of the variables was assessed using the Kolmogorov-Smirnov test. Since the assumption of normality was not met, nonparametric tests were used for subsequent analyses. Specifically, the Mann-Whitney U test was used to analyse differences in loneliness scale scores based on different categorical variables. A significance level of *p* < 0.05 was established for all statistical analyses.

## Results

A total of 112 adolescents with a mean age of 18.21 years (± 1.52) were included. With regard to the sociodemographic characteristics of the sample, it should be noted that 78% of these adolescents were between 18 and 20 years old and 69% were male (Table 1).

Almost the entire sample were students (93%). 34% were enrolled in vocational training, and 29% were enrolled in secondary school or high school (Table 1).

**Table 1:** Sociodemographic characteristics of the sample (*n* = 112)

		Frequency (%)
Age	14–17 years 18–20 years	25 (22.3%) 87 (77.7%)
Sex	Masculine Feminine	77 (68.8%) 35 (31.3%)
Academic year	Dropped out 1st year Compulsory Secondary Education 2nd year Compulsory Secondary Education 3rd year Compulsory Secondary Education 4th year Compulsory Secondary Education 1st year High School 2nd year High School Vocational education basic level Vocational education intermediate level Vocational education superior level University Work Language Centre	4 (3.6%) 0 (0) 1 (0.9%) 2 (1.8%) 8 (7.1%) 7 (6.3%) 14 (12.5%) 2 (1.8%) 20 (17.9%) 16 (14.3%) 33 (29.5%) 3 (2.7%) 1 (0.9%)
Student role	No student role Student role	6 (5.4%) 104 (92.9%)
Course repetition	No course repetition 1 year behind 2 years behind 3 or more years behind Non aplicable	74 (66.1%) 14 (12.5%) 16 (14.3%) 0 (0) 8 (7.1%)

Only 36% of the sample played every day, and 44% played less than 4 days a week. 70% played a maximum of 3 h a day, and only 11% played more than 5 h a day (Table 2). On the other hand, only 8% of adolescents had some kind of restriction on their use of videogames, and only 11% of the sample did not have their own console.

**Table 2:** Videogame usage time of the sample (*n* = 112)

		Frequency (%)
Days per week spent playing videogames	From 1 to 3 days From 3 to 5 days Everyday	49 (43.8%) 23 (20.5%) 40 (35.7%)
Hours spent in videogame use	Less than 1 h 1 h 2 h 3 h 4 h 5 h Between 5 and 7 h Between 8 and 10 h More than 10 h	7 (6.3%) 16 (14.3%) 33 (29.5%) 22 (19.6%) 14 (12.5%) 8 (7.1%) 3 (2.7%) 7 (6.3%) 2 (1.8%)

With regard to the results of the Loneliness Scale, relatively low scores were found in the study population given the scoring ranges of the tool. A range of 8 to 31 was found in the “*Peer Rejection*” area; a range of 8 to 38 in the “*Family Deficits”* and *“Parental Rejection”* area; a range of 8 to 32 in the “*Personal Inadequacy” area*; and a range of 8 to 28 in the “*Lack of Social Skills and Significant Separation*” area. In addition, a highly statistically significant difference was found in peer rejection by age group, meaning that participants in late adolescence (18–20 years) had a better perception of their friendships than adolescents in middle adolescence (14–17 years) (Table 3).

**Table 3:** Results on the adolescent loneliness scale by age group

Variable	Total *n* = 112		14–17 years *n* = 25		18–20 years *n* = 87		U Mann Whitney
	Me	IQR	Me	IQR	Me	IQR	
Peer Rejection	11.00	6	14.00	7	11.00	5	Z=-2.62, ***p*** = 0.009**
Family Deficits	14.00	11	14.00	13	14.00	11	Z = 0.07, *p* = 0.947
Personal Inadequacy	16.00	8	16.00	9	16.00	8	Z = 0.13, *p* = 0.894
Significant Separation	15.00	8	15.00	10	15.00	8	Z=-1.32, *p* = 0.188

Me = Median, IQR = Interquartile range

* Statistically significant

** High statistical significance

Results that met statistical significance were highlighted in bold within the tables

With regard to gender, a statistically significant relationship was found between gender and several areas of loneliness, indicating that women had a worse perception of their family ties, themselves, and their social skills and ability to adapt compared to men (Table 4).

**Table 4:** Relationship between loneliness and gender

Area of Loneliness	Sex				U Mann Whitney	
	Masculine		Feminine			
	Me	IQR	Me	IQR		
Peer Rejection	11.00	6	13.00	8	Z = 1.68, *p* = 0.094	
Family Deficits	12.00	9	19.00	13	Z = 2.68, ***p*** = 0.007**	
Personal Inadequacy	15.00	8	19.00	7	Z = 3.41, ***p*** = 0.001**	
Significant Separation	14.00	8	16.00	7	Z = 2.38, ***p*** = 0.017*	

Me = Median, IQR = Interquartile range

* Statistically significant

** High statistical significance

Results that met statistical significance were highlighted in bold within the tables

A statistically significant relationship was also found between daily use and the “*Area of lack of social skills and significant separation*”, which indicated that people who played 1 to 3 days had a better perception of their social and adaptive skills (Z=-2.15, *p* = 0.032); while people who played 3 to 5 days had a worse perception of them (Z = 1.98, *p* = 0.048). With regard to playing every day, no statistically significant relationship was found in any of the areas of loneliness (Table 5).

In terms of hours of use, only 9 subjects showed a statistically significant relationship between playing 8 to more than 10 h a day and the area of personal inadequacy (Z=-2.63, *p* = 0.009) (*n* = 9).

**Table 5:** Relationship between loneliness and playing every day

Area of Loneliness	Playing everyday				U Mann Whitney	
	Yes		No			
	Me	IQR	Me	IQR		
Peer Rejection	12.00	8	11.00	6	Z = 0.388, *p* = 0.698	
Family Deficits	12.00	12	15.00	11	Z=-0.774, *p* = 0.439	
Personal Inadequacy	16.00	8	17.00	7	Z=-0.155, *p* = 0.877	
Significant Separation	15.00	7	15.00	9	Z = 0.557, *p* = 0.578	

Me = Median, IQR = Interquartile range

* Statistically significant

** High statistical significance

On the other hand, adolescents who played videogames alone had a worse perception of their friendships, family ties, themselves, and their social and adaptive skills, compared to adolescents who exclusively played videogames when they were with someone else (Table 6).

**Table 6:** Relationship between loneliness and playing alone

Area of Loneliness	Playing alone				U Mann Whitney	
	Yes		No			
	Me	IQR	Me	IQR		
Peer Rejection	12.00	6	10.00	4	Z = 2.64, ***p*** = 0.008**	
Family Deficits	14.00	12	10.50	10	Z = 1.97, ***p*** = 0.048*	
Personal Inadequacy	17.00	7	14.50	7	Z = 2.07, ***p*** = 0.038*	
Significant Separation	15.50	7	12.00	5	Z = 2.74, ***p*** = 0.006**	

* Statistically significant, ** High statistical significance, Me = Median, IQR = Interquartile range

Results that met statistical significance were highlighted in bold within the tables

Likewise, no statistically significant relationships were found between areas of loneliness comparing physical co-play with online co-play (Table 7).

**Table 7:** Relationship between loneliness and videogame socialization medium

Area of Loneliness	Videogame socialization medium				U Mann Whitney
	Physical co-play		Online co-play		
	Me	IQR	Me	IQR	
Peer Rejection	11.00	4	11.00	4	Z=-0.919, *p* = 0.358
Family Deficits	13.00	8	13.00	12	Z=-0.073, *p* = 0.942
Personal Inadequacy	16.00	6	16.00	8	Z=-0.169, *p* = 0.865
Significant separation	13.00	8	15.00	6	Z=-0.962, *p* = 0.336

Me = Median, IQR = Interquartile range

* Statistically significant

** High statistical significance

## Discussion

This study aims to investigate feelings of loneliness in adolescents who attend community spaces, as well as to examine the relationship between loneliness and videogame use.

This is the first study to consider the relationship that may exist between the use of videogames within community settings and feelings of loneliness, making it novel in the preventive approach to loneliness. Although some social implications of videogames have been explored, such as the benefits of online gaming in the context of the pandemic [26, 27], the influence of social bonds established by people with autism spectrum disorder within the videogame [29], or experiences of online playing among people with spinal cord injuries [28], the social importance of playing videogames within community resources for adolescents has never been considered before.

As for loneliness in youth, although everyone can experience these feelings, they have been shown to be linked to factors such as gender, age, environmental characteristics, satisfaction with social relationships, and the existence of mental and physical health problems [7].

The results of this study show that feelings of loneliness were more pronounced in women, with a significant difference of 2 points in the *“Peer rejection” area* and *“Lack of social skills and significant separation*” area and highly statistically significant differences in the *“Family Deficits and Parental Rejection”* area of 7 points and in the *“Personal Inadequacy”* area of 4 points. These findings are consistent with previous studies indicating that 3 out of 10 young women in Spain report feeling lonely, compared to 2 out of 10 men [6].

Social experiences within gaming environments often differ between girls and boys, resulting in girls feeling discouraged from participating [35]. Combined with the present results involving loneliness in women, this suggests that gender differences should be taken into account when designing interventions related to loneliness, particularly those involving videogame activities.

In terms of age difference, adolescents aged 14 to 17 showed a more negative perception of their friendships compared to the 18 to 20 age group (Z=-2.62, *p* = 0.009). This result is consistent with the normative development of adolescence, as the need to belong to a social group fluctuates over the years and is more pronounced at younger ages [4]. This fact could manifest itself in a greater tendency to interpret social interactions more critically, and would explain their negative perception during middle adolescence.

Given that rejection by peers during adolescence causes psychological distress that can lead to serious consequences such as the development of mental health disorders, the emergence of self-harming behaviors, suicidal ideation, or suicide attempts, it would be important to specifically consider the group of adolescents between the ages of 14 and 17 in the preventive approach to loneliness [36, 37].

### Videogame usage time

The significant relationships found between the areas of loneliness and the number of days spent playing did not indicate a consistent pattern associating feelings of loneliness with more or less frequent videogame usage. In terms of hourly usage, a significant relationship was found between playing for 8 to more than 10 h and the “*Personal inadequacy*” area (Z=-2.63, *p* = 0.009), which could not be considered representative due to the sample size considered in the question (*n* = 9).

These findings are consistent with previous studies that establish inconsistent or inconclusive relationships between videogame playing time and loneliness [38]. Data that may indicate that, within this significant activity, other factors such as adolescents’ motivation to play, the quality of the social interactions they establish within the videogame, or the family or school context that surrounds them, will be more closely related to feelings of loneliness. In any case, the amount of time spent playing videogames must be taken into account as a key element for the proper development of adolescents [21, 22].

### Social environment of videogames

In our study sample, we found that adolescents who played alone reported greater loneliness, with this difference being statistically significant in the *“Family deficits and parental rejection”* area (Z = 1.97, *p* = 0.048) and *“Personal inadequacy”* area (Z = 2.07, *p* = 0.038), and highly significant in the *“Peer rejection”* area (Z = 2.64, *p* = 0.008) and “*Lack of social skills and significant separation”* area (Z = 2.74, p = 0.006). Therefore, as expected, adolescents who always played videogames exclusively with other people in community settings had better family and friendship ties, higher self-esteem, and a better perception of their social and adaptation skills, which supports the hypothesis that the use of community resources to play videogames can contribute to reducing feelings of loneliness in the adolescent population.

Furthermore, although no statistically significant differences were found within the videogame socialization environment, adolescents who played with other people in physical settings had a 2-point difference in the *“Lack of social skills and significant separation”* area, compared to adolescents that co-played predominantly online, indicating that they had a slightly better perception of their social and adaptive skills.

These results are consistent with evidence reported in multiple studies, which highlight the benefits of using videogames in community settings as a way to foster social relationships and improve mental health [12, 13, 15, 24, 39]. Although playing with other people online has also been found to have benefits on well-being indicators, such as lower rates of depression, problematic Internet use, academic stress or peer relationship stress [40]. Other studies have found that physical co-play can foster better relationships with family and friends [41–43].

In this sense, promoting access to physical community spaces that encourage adolescents to actively participate in digital leisure activities could potentially be an effective preventive strategy during this crucial stage of personal and social development.

In addition, these resources could be used with adolescents who engage in harmful use of videogames, bearing in mind that a change of environment to a community space could prevent feelings of loneliness from developing, and enhance the social characteristics associated with videogames [12, 23]. Meanwhile, from a health promotion perspective, adolescents who use community resources in their city could strengthen their social ties through videogames, which could contribute to preventing the development of behaviors linked to the onset of addiction [20].

### Limitations

This study has several limitations that should be taken into account when interpreting the findings. First, the relatively small sample size drawn through convenience sampling may restrict the external validity of the results. Second, the cross-sectional design prevents the examination of causal relationships or developmental trajectories. Third, the absence of a comparison group outside youth centres further limits the applicability of the findings to broader populations. Finally, as the majority of participants were between 18 and 20 years old, the representation of younger adolescents was limited.

### Future studies

It would be interesting to continue research on community spaces and evaluate the effectiveness of new interventions and programs based on occupational participation focused on the interests of adolescents and young people as a means of promoting health.

Likewise, it would be relevant to conduct comparative longitudinal studies between adolescents who use videogames in community spaces and those who play in private settings, in order to better understand the relationship between the context of use and loneliness. A well as differentiate between various types of co-play, explore gendered experiences of community gaming, examine the role of game genres and investigate the frequency of solitary versus social gaming for a more precise assessment of the relationship between videogame play and loneliness.

Finally, it would be advisable to develop studies that include greater representation of younger adolescents.

## Conclusions

The findings of this study show that the use of videogames within community resources could promote the development of social bonds in adolescence, contributing to a reduction in feelings of loneliness. There is a need to create specific interventions that address loneliness in a preventive manner for younger adolescents and women, as they had higher scores in the areas of loneliness.

Therefore, encouraging the use of videogames in community settings may be a useful strategy for reducing loneliness in adolescence.

## Data Availability

The datasets generated for this research are not publicly available due to their future use in ongoing studies that broaden the findings reported in the current study. However, availability of certain data can be sought form the corresponding author upon reasonable request.
